# Vitamin D Status and Multisystem Health Outcomes at 5–6 Years in Very-Low-Birth-Weight Preterm and Term-Born Children: A Cross-Sectional Secondary Analysis of Two Prospective Cohorts

**DOI:** 10.3390/nu18182994

**Published:** 2026-09-13

**Authors:** Yi-Shan Tsai, Yen-Chen Lin, Kai-Ti Tseng, Jui-Hsing Chang, Wai-Tim Jim, Chun-Chih Peng, Hung-Yang Chang

**Affiliations:** 1 Division of Neonatology, Department of Pediatrics, MacKay Children’s Hospital, Taipei 104217, Taiwan; y4131237@gmail.com (Y.-S.T.); xup6u045ptt@gmail.com (Y.-C.L.); emilyktt@gmail.com (K.-T.T.); jhchang4519@gmail.com (J.-H.C.); waitim@mmh.org.tw (W.-T.J.); pengcc4566@gmail.com (C.-C.P.); 2 Department of Medicine, MacKay Medical College, New Taipei City 251020, Taiwan

**Keywords:** vitamin D, preschool children, preterm infants, multisystem outcomes, bone mineral density, sun exposure, supplementation, physical activity

## Abstract

**Background/Objectives:** Vitamin D deficiency is common in children, but its associations with growth and organ function remain unclear. This cross-sectional secondary analysis of two prospective cohort studies evaluated vitamin D status and multisystem health outcomes in very-low-birth-weight preterm and term children aged 5–6 years. **Methods:** Serum 25-hydroxyvitamin D [25(OH)D] was categorized as deficiency (<20 ng/mL), insufficiency (20–30 ng/mL), or sufficiency (>30 ng/mL). Assessments included anthropometry, blood pressure, body composition and bone mineral density, pulmonary function, cardiac indices, kidney and metabolic biomarkers, and cognitive function (preterm children only). **Results:** Overall, 213 children (144 preterm, 69 term) were included; only 17.8% achieved sufficient vitamin D levels. No statistically significant associations were detected between serum 25(OH)D and any of these outcomes. In contrast, 25(OH)D levels were positively correlated with physical activity, sun exposure, and vitamin D supplementation (all *p* < 0.05; second cohort only). **Conclusions:** Vitamin D deficiency was common, but no statistically significant associations with multisystem health measures were detected; several features of this analysis limit the strength of these null findings. Sun exposure, physical activity, and vitamin D supplementation were associated with higher 25(OH)D concentrations. These exploratory cross-sectional findings require confirmation in prospectively designed studies.

## 1. Introduction

Vitamin D regulates calcium and phosphorus absorption, supporting bone mineralization and skeletal growth. However, increasing evidence demonstrates that vitamin D has pleiotropic physiological effects beyond bone health [1,2]. Vitamin D influences multiple organ systems, including the immune, nervous, musculoskeletal, and cardiovascular systems [1]. Its functions include immune response modulation, support of cardiopulmonary development, maintenance of vascular and endothelial function, and key contributions to neurocognitive processes. These integrated mechanisms highlight the importance of adequate vitamin D status for metabolic regulation and healthy childhood development [2,3].

Vitamin D status is typically assessed by serum 25-hydroxyvitamin D (25(OH)D), the major circulating metabolite and most reliable indicator of body stores. Despite increased recognition of its importance, vitamin D deficiency remains a substantial global health concern. Systematic reviews estimate that 30–50% of newborns, children, and adolescents worldwide exhibit insufficient or deficient 25(OH)D levels [2,4,5,6]. The etiology is multifactorial and includes insufficient ultraviolet B exposure, inadequate dietary intake, obesity-related adipose tissue sequestration, and genetic, cultural, and environmental factors [2]. In children, suboptimal vitamin D levels have been clinically linked to impaired bone mineralization, rickets, growth disturbances, recurrent respiratory infections, asthma, and metabolic dysregulation [7,8,9,10,11]. Furthermore, preterm infants are particularly vulnerable owing to limited transplacental transfer, reduced cutaneous synthesis, immature hepatic activation, and frequent nutritional inadequacies in early postnatal life [12,13].

Despite growing awareness of these health implications, few studies have evaluated vitamin D status in preschool-aged children, a developmental period characterized by rapid growth, organ maturation, and evolving metabolic demands-including skeletal mineralization, pulmonary and cardiovascular development, and the consolidation of metabolic regulation-that follows the rapid catch-up growth of infancy and immediately precedes school entry [3,14,15,16,17,18,19,20,21,22]. Understanding whether vitamin D status at 5–6 years is linked to multisystem health outcomes may inform pediatric nutritional strategies. Additionally, behavioral factors such as sun exposure, physical activity, and supplementation may strongly influence vitamin D levels. The aim of this study was to examine associations between serum 25(OH)D concentrations and growth, body composition and bone mineral density, pulmonary and cardiovascular function, kidney and metabolic biomarkers among term and very-low-birth-weight (VLBW) preterm children aged 5–6 years, and cognitive function in the preterm group.

## 2. Materials and Methods

### 2.1. Study Population

This study was a secondary analysis of two previous prospective cohort studies involving preterm and term control children aged 5–6 years. Because serum 25(OH)D and the outcome measures were obtained at the same assessment visit at 5–6 years, the present analysis is cross-sectional in design. Preterm children born at gestational age (GA) < 37 weeks with birth weight (BW) < 1500 g and followed at our institution’s preterm follow-up clinic were invited to participate. Term-born controls (GA ≥ 37 weeks, BW > 2500 g) were recruited from the hospital’s outpatient clinics and inpatient wards, with recruitment continued until the group-level age distribution approximated that of the preterm group; individual matching was not performed. In the first cohort (from 1 April 2019–31 March 2021), preterm children and term-born controls were prospectively recruited to evaluate lung [23] (IRB No. 16MMHIS162e) and cardiovascular function [24] (IRB No. 17MMHIS037e). In the second cohort (from 1 January 2021–31 December 2022), preterm and term-born children were enrolled to assess kidney function [25] (IRB No. 19MMHIS235e) and body composition [26] (IRB No. 19MMHIS261e). Participants who declined blood sampling were excluded from the present analysis. The current study received approval from the Institutional Review Board at MacKay Memorial Hospital (Approval No. 25MMHIS525e).

### 2.2. Serum 25(OH)D Level

Serum 25(OH)D measurements were performed concurrently with the multisystem outcome evaluations at age 5–6 years. Serum 25(OH)D was not part of either original cohort protocol; it was measured at the same visit as the other outcome assessments. Fasting for at least 8 h, confirmed by parental report, was required before blood sampling in both cohort studies. Serum 25(OH)D levels were analyzed using a chemiluminescence method (LIAISON XL; DiaSorin S.p.A., Saluggia, Italy). Measurements were performed through the hospital’s accredited clinical chemistry laboratory under routine internal quality control procedures.

Vitamin D status was classified based on serum 25(OH)D levels into three categories: deficiency (<20 ng/mL), insufficiency (20–30 ng/mL), and sufficiency (>30 ng/mL).

Other assessments and laboratory methods were detailed in previous studies and are briefly summarized here.

### 2.3. Anthropometric and Blood Pressure (BP) Measurements

All participants underwent anthropometric and BP measurements at 5–6 years of age. Height and weight were measured using a digital scale and converted to age- and sex-independent z-scores. Participants were categorized as underweight, normal weight, overweight, or obesity using age- and sex-specific body mass index (BMI) criteria for Taiwanese children adapted from World Health Organization guidelines [27]. BP was measured using a Dinamap device (DPC120X-EN; GE Medical Systems, Milwaukee, WI, USA) with an appropriately sized cuff, with the child seated and at rest. Three readings were taken at 1–2 min intervals during the same visit, and the mean of the three was used for analysis.

### 2.4. Body Composition and Bone Densitometry Measurements

Participants in the second cohort underwent body composition and bone densitometry assessment using dual-energy X-ray absorptiometry (DXA) (Horizon DXA System, APEX software version 5.6; Hologic Inc., Marlborough, MA, USA). The DXA system underwent routine quality control and phantom calibration in accordance with the manufacturer’s recommendations throughout the study period. All scans were performed by a single pediatric densitometry specialist blinded to participants’ clinical status, using standardized pediatric positioning and region-of-interest placement. Total lean mass index, fat mass index, fat distribution across the trunk, total body less head, and whole-body composition were evaluated. Bone mineral content (BMC, g) and areal bone mineral density (BMD, g/cm^2^) were assessed at the total body less head (TBLH), the site recommended for pediatric densitometry, and standardized BMD z-scores were derived from the Hologic international reference database, which adjusts for age and sex.

### 2.5. Lung Function

Participants from the first cohort underwent spirometry testing (Ultima PF with RTD; MGC Diagnostics, Saint Paul, MN, USA) to obtain spirometric parameters, including forced expiratory volume in one second (FEV_1_), forced vital capacity (FVC), FEV_1_/FVC ratio, and forced expiratory flow between 25% and 75% of expired FVC (FEF_25–75_). Lung function results were expressed as z-scores adjusted for height, sex, age, and race, based on reference values from the Global Lung Function Initiative [28]; Taiwan-specific validation of these equations was not available.

### 2.6. Echocardiographic Measurements

Participants in the first cohort underwent echocardiographic assessment using two-dimensional imaging, M-mode imaging, and two-dimensional speckle-tracking echocardiography (2DSTE) to evaluate cardiac anatomy and myocardial function. Examinations were performed using a Vivid system (GE Vingmed Ultrasound AS, Horten, Norway) with a 2–4 MHz transducer. Left atrial and left ventricular myocardial deformation parameters, including longitudinal peak systolic strain, endocardial velocity, global longitudinal strain, and peak left atrial longitudinal strain, were analyzed offline using EchoPAC software version 10.8 (GE Vingmed Ultrasound AS, Horten, Norway).

### 2.7. Kidney Function

Participants in the second cohort underwent kidney function assessments, including kidney ultrasound and blood testing. The glomerular filtration rate was estimated (eGFR) using the updated CKiD U25 equation based on serum cystatin C, serum creatinine, and the average of both estimates [29], which, although originally derived and validated in children and young adults with chronic kidney disease, is now a widely adopted standard for eGFR estimation in pediatric populations more broadly.

### 2.8. Biochemical Analysis

In addition to vitamin D and kidney function assessments, serum samples from the second cohort were analyzed for glucose, albumin, alkaline phosphatase, lipids, minerals, and insulin. Insulin resistance was estimated using the homeostatic model assessment of insulin resistance (HOMA-IR), calculated as (fasting glucose (mg/dL) × insulin (µU/mL))/405.

### 2.9. Questionnaire

In the first cohort, parents completed a questionnaire on their child’s respiratory morbidity and atopy history. The questionnaire was based on the modified Chinese version of the International Study of Asthma and Allergies in Childhood questionnaire [30]; the present analysis draws on the physician-diagnosed asthma and lower respiratory tract infection rehospitalization items from this instrument. Physician-diagnosed asthma was defined by regular use of asthma medications, including inhaled corticosteroids, beta-2-agonists, or anti-leukotriene agents, within the preceding 12 months.

In the second cohort, parents reported the vitamin D supplementation status, weekly duration of sun exposure, and physical activity of their child. These items were completed by parents or legal guardians as part of the Cohort 2 questionnaire and asked about typical weekly duration at the time of the assessment visit; they were not drawn from a previously validated standalone instrument. Continuous vitamin D supplementation was defined as intake of ≥400 IU/d for at least 6 months [31]; specific dose, formulation, and exact duration beyond this threshold were not captured. Across both cohorts, parents reported hospitalizations for lower respiratory tract infections, including bronchiolitis and pneumonia.

### 2.10. Cognition Outcomes

Cognitive assessment was exclusively performed in preterm children, as neurodevelopmental follow-up is part of the standard clinical protocol for VLBW preterm survivors at our institution. Therefore, term-born controls were not cognitively assessed. Cognitive abilities were assessed at 5 years of age in 137 of 144 preterm children using the Chinese version of the Wechsler Preschool and Primary Scale of Intelligence, Fourth Edition (WPPSI-IV) [32], normed for children aged 2.5 to 7.5 years and therefore age-appropriate for the present assessment, administered by an experienced, qualified clinical psychologist at the same visit as blood sampling. The examining psychologist was blinded to vitamin D status, as biochemical results were not available to clinical staff at the time of testing. The remaining seven children were excluded, as they had been evaluated using alternative cognitive assessment methods. General cognitive ability was measured using the full-scale intelligence quotient and was further divided into five component indices: Verbal Comprehension Index, Visual Spatial Index, Fluid Reasoning Index, Processing Speed Index, and Working Memory Index.

### 2.11. Statistical Analyses

Serum 25-hydroxyvitamin D [25(OH)D] levels were compared across predefined host-related subgroups, including prematurity, sex, small for gestational age (SGA), BMI status, and vitamin D supplementation. Continuous 25(OH)D levels were analyzed using independent-samples t tests for binary subgroup comparisons and one-way analysis of variance (ANOVA) for BMI categories. To evaluate potential seasonal bias in vitamin D measurement, serum 25(OH)D concentrations were compared across four seasons using one-way ANOVA: spring, March–May; summer, June–August; autumn, September–November; and winter, December–February. Health outcomes across vitamin D categories (sufficiency, insufficiency, and deficiency) were compared using one-way ANOVA for continuous variables and chi-square tests for categorical variables. Serum 25(OH)D was adjusted for age, sex, GA, SGA status, and season of blood sampling. BW was not additionally adjusted for, given its collinearity with GA. Outcomes already standardized for age and sex (z-score-based outcomes, eGFR, and BMD z-score) were adjusted for GA, SGA, and season only, to avoid overadjustment. Season was entered as three indicator variables with winter as the reference category. Adjusted *p*-values for continuous outcomes were obtained from multivariable linear regression, and for categorical outcomes from logistic regression. Cohort membership was included as an additional covariate only for the two outcomes assessed in both cohorts (anthropometry, blood pressure); for all other outcome domains, cohort membership is constant and was not applicable.

Associations between serum 25(OH)D levels and selected health indicators were examined using Spearman age- and sex-adjusted partial correlation analyses. Partial correlations were computed by regressing each rank-transformed variable on the covariate set and correlating the residuals (equivalent to a Spearman-based partial correlation). Distributional assumptions were assessed using the Shapiro–Wilk test; triglycerides, insulin, and HOMA-IR were right-skewed and were log-transformed before regression. Log transformation did not improve the distribution of plasma renin activity, which was therefore analyzed on the original scale using the Kruskal–Wallis test for group comparisons and Spearman correlation for association analyses. Homoscedasticity and influential observations were assessed via residual diagnostics and Cook’s distance.

All tests were two-sided, and *p* < 0.05 was considered statistically significant. Statistical analyses were conducted using SPSS Statistics, version 26.0 (IBM Corp., Armonk, NY, USA). All analyses were complete-case; missing data were not imputed.

Because no vitamin D-related primary or secondary outcome was prespecified in either parent cohort protocol, all analyses in this study are exploratory. The Benjamini–Hochberg procedure was used to control the false discovery rate at 5% within each outcome domain (anthropometry/growth, body composition, bone mineral density, cardiopulmonary, biochemical/renal, cognitive, and questionnaire-based), applied separately to the categorical and continuous exposure analyses.

## 3. Results

### 3.1. Participant Characteristics

**Figure 1:**
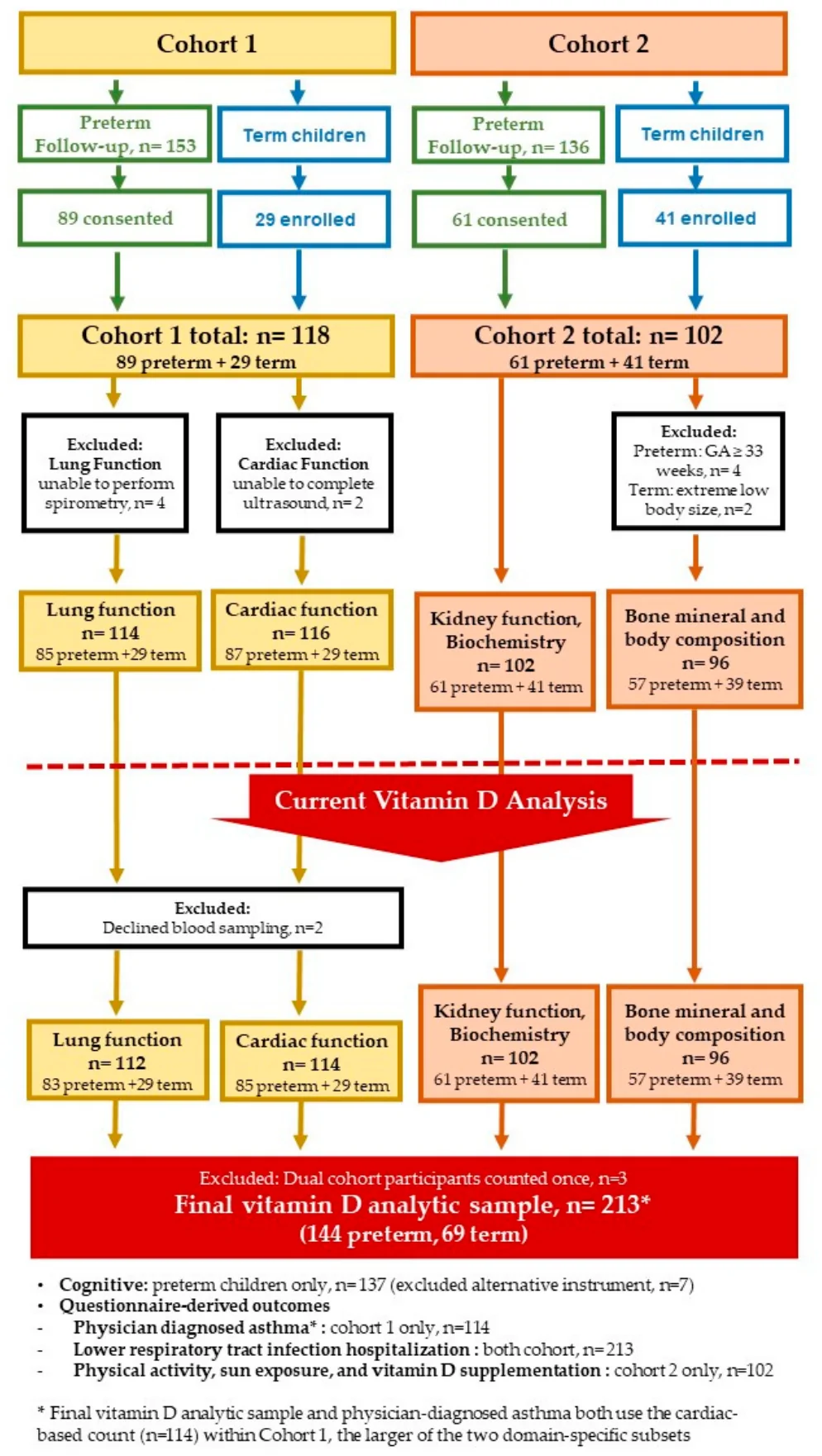
Flow diagram of participant inclusion in the current vitamin D analysis and related sub-studies. Green and blue boxes denote the preterm and term-born arms, respectively; yellow and orange shading denote Cohort 1 and Cohort 2. The red dashed line separates the original sub-study recruitment (above) from the present vitamin D analysis (below).

Figure 1 presents the study flow diagram for participant inclusion in the current analysis and related studies.

For Cohort 1, 153 children were invited from the preterm follow-up program and 89 consented. Within this group, 85 children had lung function data (4 excluded for inability to reliably perform spirometry) and 87 had cardiac data (2 excluded for inability to complete echocardiography) [23,24]. A further 2 children who had completed these sub-study assessments declined blood sampling for the present analysis, yielding 83 preterm children with both lung function and 25(OH)D data, and 85 preterm children with both cardiac data and 25(OH)D data. The larger of these two subsets (85 preterm, plus 29 term-born controls) is used as the Cohort 1 total (*n* = 114) for the combined analytic sample.

For Cohort 2, 136 children were invited and 61 consented [26], with no additional exclusions for blood sampling; the second cohort included 102 children: 61 preterm and 41 term-born controls. Term-born controls were recruited opportunistically from outpatient clinics and inpatient wards rather than from a defined registry, and no comparable eligible-population figure exists for this arm.

Assessments included lung function (*n* = 112), cardiac function (*n* = 114), kidney function (*n* = 102), and body composition and bone mineral density (*n* = 96). Six Cohort 2 participants did not undergo DXA assessment, having been excluded under the DXA sub-study inclusion criteria (4 preterm children born at ≥33 weeks’ gestation; 2 term-born children with extreme low body size), consistent with the analytic sample sizes reported below. Three preterm participants were enrolled in all four sub-studies. Differences in analytic sample size across outcome domains reflect the cohort-specific assessment protocols rather than item-level missing data; individual-level exclusions are shown in Figure 1. Combining the Cohort 1 total (*n* = 114) and Cohort 2 total (*n* = 102) and counting the three dual-cohort participants once yielded a final analytic sample of 213 participants, consisting of 144 preterm and 69 term-born children.

Among preterm participants, mean GA and BW were 28.6 ± 2.7 weeks (range 23.0–36.0) and 1055 ± 256 g (range 530–1496), respectively. In term-born controls, mean GA and BW were 38.6 ± 1.1 weeks (range 37.0–41.0) and 3048 ± 335 g (range 2324–3970), respectively. Mean assessment age was 5.5 ± 0.5 years in the preterm group and 5.9 ± 0.6 years in the term group. Among the full VLBW preterm sample followed in our program during the study period (*n* = 286), the 144 children enrolled in the present analysis did not differ significantly from the 142 not enrolled in any of 18 perinatal or neonatal characteristics, including gestational age, birth weight, and major neonatal morbidities (all *p* ≥ 0.067; Table S1).

Cohort 1 and Cohort 2 differed significantly in the proportion of patients born preterm (75.4% vs. 59.8%, *p* = 0.021), gestational age (31.1 ± 4.9 vs. 32.6 ± 5.4 weeks, *p* = 0.030), birth weight (1522 ± 888 vs. 1877 ± 1038 g, *p* = 0.008), season of blood sampling (*p* = 0.015), and serum 25(OH)D concentration (24.3 ± 5.9 vs. 22.5 ± 6.9 ng/mL, *p* = 0.038), but not in age at assessment (*p* = 0.825) or sex (*p* = 1.000) (Table S2). Effect sizes for these differences were small (Cohen’s d = 0.29–0.37; Table S3), and mean 25(OH)D in both cohorts fell within the same insufficiency category (20–30 ng/mL). Because each outcome domain was assessed within, not across, cohorts, these between-cohort differences do not confound the outcome-specific analyses presented below.

Two sets of interaction analyses were performed to assess whether pooled analysis was appropriate. A 25(OH)D × prematurity interaction term was tested for each of 23 principal outcomes; no interaction reached statistical significance (all *p* ≥ 0.09). Gestational age was omitted from the covariate set in these models because it is collinear with preterm status, and cognitive outcomes could not be tested because cognitive assessment was restricted to preterm children. A 25(OH)D × cohort interaction was tested for the two outcome domains assessed in both cohorts (anthropometry, blood pressure); no interaction was statistically significant (all *p* ≥ 0.12; *n* = 210, excluding the three participants enrolled in both cohorts, for whom cohort membership cannot be uniquely assigned). These results support analysis of the pooled sample with adjustment for gestational age and SGA status.

### 3.2. Host-Related Factors Associated with Vitamin D Level and Status

**Figure 2:**
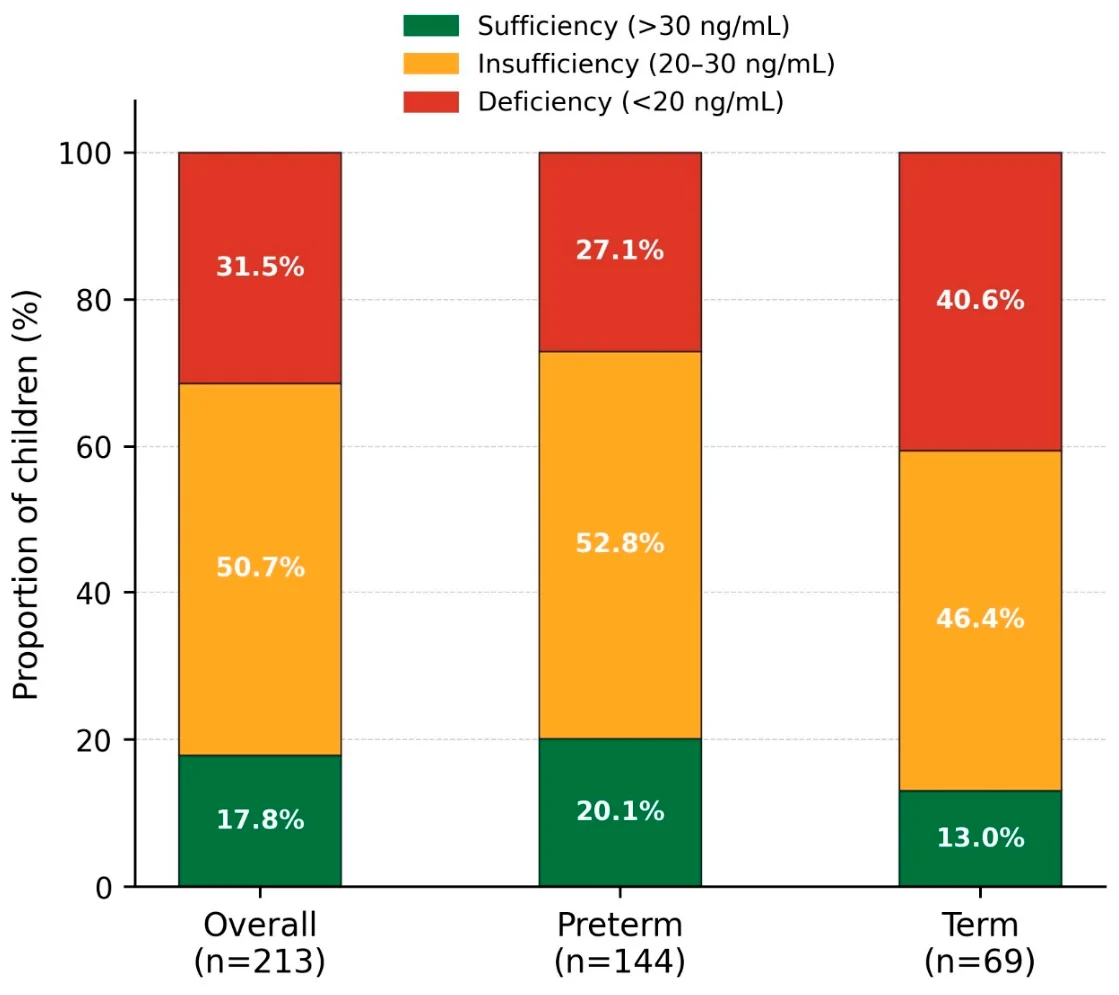
Vitamin D status distribution across the overall cohort, preterm children, and term children.

The overall cohort had a mean serum 25(OH)D concentration of 23.4 ± 6.4 ng/mL. Vitamin D insufficiency was present in 50.7% (108/213) of participants, and deficiency in 31.5% (67/213), whereas only 17.8% (38/213) had sufficient levels (Figure 2). The proportion of children with deficiency was 40.6% in term-born and 27.1% in preterm children (*p* = 0.053; Figure 2).

Blood samples were collected across all four seasons, with 25.4%, 28.6%, 28.6%, and 17.4% of participants sampled in spring, summer, autumn, and winter, respectively. Serum 25(OH)D concentrations did not differ significantly across seasons (spring: 22.6 ± 6.6, summer: 23.7 ± 6.3, autumn: 23.9 ± 6.4, winter: 22.9 ± 6.5 ng/mL; *p* = 0.632). This should be read as an absence of a detected seasonal difference rather than definitive evidence against seasonal variation.

Table 1 shows that mean serum 25(OH)D concentrations did not differ significantly between term and preterm children, between boys and girls, or between appropriate-for-gestational-age and SGA children. Likewise, mean 25(OH)D concentration did not differ significantly across BMI categories. In contrast, children receiving vitamin D supplementation had significantly higher serum 25(OH)D concentrations than those without supplementation.

### 3.3. Health Outcomes Across Different Vitamin D Categories

Participants were stratified into vitamin D sufficiency (*n* = 38), insufficiency (*n* = 108), and deficiency (*n* = 67) groups based on serum 25(OH)D concentrations. Table 2 compares baseline characteristics and multisystem health outcomes across the three groups.

#### 3.3.1. Basic Characteristics

No significant differences in GA, BW, prematurity, or SGA status were observed among the vitamin D groups. However, sex distribution varied significantly across groups, with boys overrepresented in the vitamin D sufficiency group (68.4%) and underrepresented in the insufficiency group (42.6%). Given the modest size of the sufficiency group (*n* = 38), this imbalance is most plausibly a chance finding rather than a biologically meaningful association between sex and vitamin D status. Age at examination did not differ significantly among groups (*p* = 0.071). Anthropometric indices, including height, weight, and BMI z-scores, did not differ significantly. The prevalence of overweight/obesity was also similar among the three groups.

#### 3.3.2. Health Outcomes at Preschool Age

Bone mineral outcomes were assessed by DXA in 96 children from the second cohort. TBLH bone mineral content differed across vitamin D categories before adjustment (385.2 ± 67.7, 400.1 ± 47.7, and 424.7 ± 61.7 g in the sufficiency, insufficiency, and deficiency groups respectively; *p* = 0.047), but this difference was not significant after covariate adjustment (*p* = 0.178). Neither TBLH bone mineral density (*p* = 0.433 unadjusted, 0.838 adjusted) nor the standardized BMD z-score (*p* = 0.514 unadjusted, 0.638 adjusted) differed across groups.

Lung function, blood pressure, and cardiac function, assessed using conventional echocardiography and two-dimensional speckle-tracking echocardiography (2DSTE), were similar across the three vitamin D groups. Body composition, kidney function, and serum biochemical profiles showed no statistically significant between-group differences.

Cognitive outcomes among preterm children were comparable across groups. Questionnaire-based outcomes showed no statistically significant association between vitamin D status and asthma or hospitalization for lower respiratory tract infection. In the second cohort, where these variables were available (*n* = 102), weekly durations of physical activity and sun exposure were the only outcome variables to differ significantly among the groups after adjustment.

### 3.4. Correlation of Vitamin D Level and Related Health Outcomes (Table 3)

**Figure 3:**
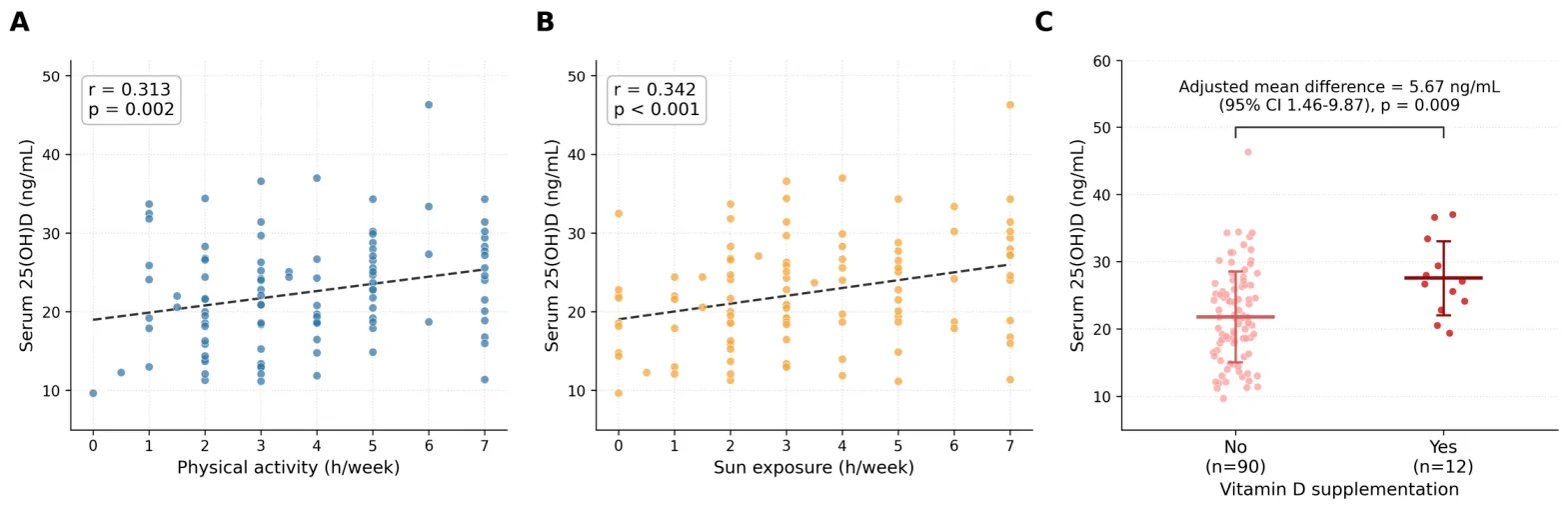
Associations between serum 25(OH)D concentrations and modifiable lifestyle factors among children in the second cohort (*n* = 102). (**A**) Weekly physical activity duration; (**B**) weekly sun exposure duration; (**C**) vitamin D supplementation status. Scatter plots (panels (**A**,**B**)) display individual data points with unadjusted regression lines; reported r values represent age-, sex-, gestational age-, SGA-, and season-adjusted partial correlation coefficients. Panel C displays individual data points for both groups alongside the group means and standard deviations; the reported adjusted mean difference and *p* value are derived from the same covariate-adjusted model used throughout this manuscript.

Serum 25(OH)D concentration was weakly inversely correlated with age at examination. Serum 25(OH)D concentration showed no significant correlations with most cardiopulmonary, metabolic, kidney, body composition, bone mineral density, or cognitive variables. Although a weak inverse correlation with left ventricular end-diastolic volume was observed in unadjusted analyses (r = −0.186, *p* = 0.048), this association was not significant after covariate adjustment (*p* = 0.072). In contrast, weekly physical activity (r = 0.313, adjusted *p* = 0.002) and sun exposure (r = 0.342, adjusted *p* < 0.001) remained positively associated with serum 25(OH)D concentration (Figure 3), and children receiving vitamin D supplementation had concentrations 5.67 ng/mL higher than non-users after adjustment (95% CI 1.46–9.87, *p* = 0.009).

After Benjamini–Hochberg correction within each outcome domain, the associations of serum 25(OH)D with sun exposure (q = 0.002), physical activity (q = 0.003), and vitamin D supplementation (q = 0.009) remained significant when 25(OH)D was analyzed as a continuous exposure; no multisystem health outcome survived correction (Table S4).

In a sensitivity analysis dichotomizing 25(OH)D at 20 ng/mL (≥20 ng/mL, *n* = 146; <20 ng/mL, *n* = 67), physical activity (adjusted mean difference 0.98 h/week, 95% CI 0.21–1.76) and sun exposure (0.87 h/week, 95% CI 0.01–1.72) remained significantly associated with vitamin D status, and no multisystem health outcome showed a materially different result (Table S5).

Physical activity and sun exposure were strongly correlated (r = 0.747, *p* < 0.001). When both were entered into the same multivariable model together with age, sex, and season (*n* = 102), variance inflation factors were 2.37 and 2.36 respectively, indicating no problematic collinearity. Supplement users (*n* = 12) and non-users (*n* = 90) did not differ significantly in prematurity status, GA, BW, SGA status, sex, age at assessment, BMI z-score, or season of blood sampling (Table S6).

**Table 3:** Correlations Between Serum 25(OH)D Levels and Selected Health Indicators.

	N	r (Unadjusted)	p (Unadjusted)	r (Partial)	β Per 10 ng/mL (95% CI)	Covariate-Adjusted *p*-Value
Age at examination	213	−0.161	0.019	–	–	–
Height z-score	213	−0.117	0.089	−0.075	−0.137 (−0.323, 0.049)	0.282
Weight z-score	213	−0.131	0.056	−0.095	−0.147 (−0.390, 0.095)	0.170
BMI z-score	213	−0.071	0.303	−0.048	−0.068 (−0.373, 0.238)	0.492
Total lean mass index (g/m^2^)	96	0.001	0.995	−0.050	58 (−209, 324)	0.643
Total fat mass index (g/m^2^)	96	−0.082	0.427	−0.062	195 (−190, 581)	0.562
Trunk fat (%)	96	−0.125	0.226	−0.096	0.11 (−1.35, 1.56)	0.370
Total fat (%)	96	−0.123	0.233	−0.111	0.05 (−1.22, 1.33)	0.300
TBLH BMC (g)	96	−0.210	0.040	−0.167	−13.62 (−29.61, 2.37)	0.118
TBLH BMD (g/cm^2^)	96	−0.051	0.619	−0.002	−0.001 (−0.013, 0.011)	0.987
TBLH BMD z-score	96	−0.020	0.849	0.024	0.025 (−0.295, 0.345)	0.820
FEV1 z-score	112	0.032	0.734	0.086	0.125 (−0.231, 0.480)	0.377
FVC z-score	112	0.061	0.525	0.104	0.184 (−0.192, 0.560)	0.284
FEV1/FVC z-score	112	−0.150	0.113	−0.137	−0.144 (−0.480, 0.192)	0.160
FEF25-75 z-score	112	−0.048	0.617	−0.010	−0.037 (−0.400, 0.326)	0.915
Systolic BP (mmHg)	213	−0.100	0.146	−0.077	−1.16 (−3.34, 1.01)	0.275
Diastolic BP (mmHg)	213	−0.041	0.550	−0.099	−1.31 (−2.94, 0.32)	0.159
LVEDV (ml)	114	−0.186	0.048	−0.175	−2.22 (−4.85, 0.42)	0.072
LVESV (ml)	114	−0.160	0.089	−0.150	−0.73 (−1.66, 0.19)	0.122
Stroke volume (ml)	114	−0.174	0.064	−0.163	−1.48 (−3.55, 0.58)	0.094
Shortening fraction (%)	114	−0.068	0.473	−0.081	−0.40 (−1.80, 0.99)	0.405
LV global longitudinal strain (%)	114	0.047	0.617	0.009	−0.079 (−0.590, 0.432)	0.923
LA longitudinal strain (%)	114	0.011	0.911	0.071	0.62 (−1.49, 2.73)	0.465
Cr (mg/dL)	102	0.030	0.767	0.005	0.001 (−0.019, 0.021)	0.959
Cystatin C (mg/L)	102	−0.026	0.795	−0.092	−0.014 (−0.056, 0.028)	0.377
eGFR-Cr (mL/min/1.73 m^2^)	102	−0.094	0.348	−0.046	−0.09 (−4.67, 4.50)	0.655
eGFR-CysC (mL/min/1.73 m^2^)	102	0.044	0.663	0.112	0.88 (−3.19, 4.96)	0.274
Calcium (mg/dL)	102	0.113	0.256	0.111	0.040 (−0.036, 0.116)	0.285
Phosphate (mg/dL)	102	−0.100	0.316	−0.103	−0.079 (−0.207, 0.048)	0.319
Alkaline Phosphatase (IU/L)	102	−0.122	0.222	−0.168	−13.35 (−27.13, 0.44)	0.104
Insulin (uU/mL)	102	−0.023	0.820	−0.072	−0.051 (−0.241, 0.139) [log scale]	0.486
HOMA-IR index	102	−0.043	0.670	−0.095	−0.061 (−0.261, 0.140) [log scale]	0.360
Full-Scale Intelligence Quotient (FSIQ) *	137	−0.124	0.149	−0.104	−2.37 (−5.34, 0.60)	0.241
Physical activity (hours/week)	102	0.256	0.009	0.313	0.89 (0.34, 1.43)	**0.002**
Sun exposure (hours/week)	102	0.294	0.003	0.342	1.08 (0.50, 1.67)	**<0.001**
Vitamin D supplementation	102	–	0.006	–	Adj. mean diff.: 5.67 (1.46, 9.87) ng/mL	**0.009**

BMI, body mass index; TBLH, total body less head; BMC, bone mineral content; BMD, bone mineral density; FEV_1_, forced expiratory volume in the first second; FVC, forced vital capacity; FEF_25–75_, forced expiratory flow 25–75%; LVEDV, left ventricular end-diastolic volume; LVESV, left ventricular end-systolic volume; LV, left ventricle; LA, left atrium; Cr, creatinine; eGFR, estimated glomerular filtration rate; HOMA-IR, Homeostasis Model Assessment of Insulin Resistance. * Preterm children only. Partial correlation = rank-transformed variables residualized on covariates (Spearman-equivalent). β = adjusted linear regression coefficient per 10 ng/mL increase in serum 25(OH)D. Standardized (z-score/eGFR/BMD-z) outcomes adjusted for gestational age (GA) + small for gestational age (SGA) + season; all other outcomes adjusted for age + sex + GA + SGA + season; systolic/diastolic BP additionally adjusted for cohort. Insulin and HOMA-IR β are on the log scale (geometric mean ratio per 10 ng/mL: Insulin 0.950 [95% CI 0.785–1.149]; HOMA-IR 0.941 [0.770–1.150]). Vitamin D supplementation is a binary variable and is reported as an adjusted mean difference in 25(OH)D rather than a correlation coefficient. Bold/shaded = *p* < 0.05. False discovery rate q-values for all comparisons are reported in Supplementary Table S4.

## 4. Discussion

Our results demonstrated a high prevalence of vitamin D insufficiency and deficiency among preschool-aged children. However, no statistically significant associations were detected between vitamin D status or serum 25(OH)D concentrations and growth, body composition, bone mineral density, pulmonary function, cardiovascular structure and function, or kidney and metabolic markers. Similarly, no statistically significant associations were detected with cognitive outcomes in preterm children. These null findings align with a growing body of pediatric evidence in which mild-to-moderate variation in vitamin D status has not been consistently associated with multisystem physiological outcomes in otherwise healthy children [1,2,3,9,13]. In contrast, modifiable lifestyle factors, including vitamin D supplementation, physical activity, and sun exposure, were significantly associated with serum 25(OH)D concentration in this cohort. Several features of this analysis limit the strength of these null findings and should be borne in mind throughout the discussion below: the generally healthy survivor population, the cross-sectional design based on a single 25(OH)D measurement, the modest size of several outcome-specific subsamples, the relatively narrow range of 25(OH)D concentrations observed, and unrecorded covariates that preclude excluding residual confounding. These findings therefore reflect the statistical precision available in each domain rather than evidence that vitamin D lacks physiological relevance. Given the exploratory nature of this analysis and the number of outcome domains evaluated, findings should be interpreted as hypothesis-generating rather than confirmatory.

Compared with a large-scale Taiwanese pediatric cohort study conducted a decade ago [5], the prevalence appeared lower; however, this comparison should be interpreted cautiously given the difference in age distribution between the two studies, which was not formally adjusted for. We observed no significant sex-based differences in serum 25(OH)D concentrations, consistent with prior reports in preschool-aged populations [14,33]. Although preterm infants are at an increased biological risk of vitamin D deficiency in early life, preterm participants at preschool age had higher mean 25(OH)D concentrations than term peers, although this difference was not statistically significant. This pattern may reflect continued nutritional supplementation and closer clinical follow-up through the preterm follow-up program; alternatively, term-born controls, perceived as lower-risk, may have been less likely to receive supplementation than preterm children under structured follow-up. Both explanations remain speculative and cannot be distinguished with the present data.

An inverse association between 25(OH)D concentrations and BMI has been widely reported in children, with vitamin D deficiency more common in those with obesity than in healthy controls [8,34,35,36,37]. Proposed mechanisms include vitamin D regulation of adipogenesis and vitamin D-metabolizing enzymes [34]. Behavioral factors may also contribute, as children with obesity often engage in fewer outdoor activities, limiting sun exposure and vitamin D synthesis [2]. In contrast, no statistically significant differences in 25(OH)D concentrations or vitamin D status were detected across BMI categories in our cohort, and serum 25(OH)D was not significantly correlated with BMI. The lack of an association in our cohort may suggest that adiposity-related effects on vitamin D metabolism are less pronounced in preschool-aged children, potentially because underlying mechanisms, including adipose tissue sequestration, reduced outdoor activity, or hormonal changes, may require a longer duration of excess adiposity to become clinically detectable.

Vitamin D is essential for skeletal health, facilitating intestinal calcium absorption and adequate mineralization. Because the skeleton is the organ system most directly regulated by vitamin D, we additionally examined DXA-derived bone mineral outcomes in the Cohort 2 subgroup (*n* = 96). TBLH bone mineral content differed across vitamin D categories before adjustment but not after covariate adjustment, and neither bone mineral density nor the age- and sex-standardized BMD z-score differed at any stage. Absolute densitometric measures in growing children are strongly influenced by age and body size, and their interpretation therefore depends on appropriate adjustment or standardization [19]. One possibility is that the skeletal consequences of vitamin D status accumulate over a longer period than the interval captured here and had not yet become detectable at 5–6 years of age.

Although experimental studies suggest that vitamin D may exert pleiotropic cardioprotective effects through modulation of nuclear factor kappa B signaling, suppression of the renin–angiotensin system, and activation of vitamin D receptors in cardiomyocytes and vascular endothelial cells, clinical evidence remains inconsistent [10,38,39,40,41]. Observational studies have reported potential associations between vitamin D status and blood pressure regulation [42]. However, meta-analyses have yielded conflicting results regarding the antihypertensive effects of vitamin D supplementation [42,43]. In our cohort, no association with blood pressure or cardiac structure and function was detected in this subsample (*n* = 114), in which blood pressure and echocardiographic measures were largely within the normal range.

Animal studies suggest that vitamin D deficiency may adversely affect lung development, including alveolarization, vascular development, and subsequent pulmonary function [12,44]. Systematic reviews in humans have reported positive associations between 25(OH)D concentrations and pulmonary function parameters, increasing interest in the potential role of vitamin D in asthma management [7]. However, clinical evidence remains inconsistent, as epidemiological associations between low 25(OH)D levels and increased asthma prevalence, particularly in children, are not consistently supported by interventional trials or meta-analyses [45,46,47]. In the present cohort, vitamin D status was not significantly associated with spirometric lung function indices or physician-diagnosed asthma. Given the conflicting evidence in the literature and the modest size of this subsample (*n* = 112), the present findings neither support nor exclude an association between vitamin D status and respiratory outcomes at this age.

Vitamin D plays an important role in both innate and adaptive immunity through the binding of its active form, 1,25-dihydroxyvitamin D (1,25(OH)_2_D), to vitamin D receptors expressed on T and B lymphocytes, monocytes, and macrophages [48]. This interaction promotes antimicrobial peptide production and may inhibit viral replication, enhancing pathogen clearance and modulating inflammatory responses [49]. Although an observational study has reported inverse associations between serum 25(OH)D levels and upper respiratory tract infections in school-aged children [50], evidence from preschool populations and randomized controlled trials has not demonstrated consistent protective effects [14,16,51]. Similarly, we did not observe a significant association between vitamin D status and hospitalization for lower respiratory tract infections in the present cohort. This finding aligns with the most recent individual participant data meta-analysis of vitamin D supplementation for acute respiratory infection prevention, which identified no overall benefit among children [51]. Together with the existing trial evidence, these results indicate that any effect of vitamin D on infection-related hospitalization in early childhood, if present, was not detectable in this cohort.

The kidneys are the primary site for conversion of 25(OH)D to 1,25(OH)_2_D, and impaired glomerular filtration may disrupt this metabolic pathway and potentially reduce circulating vitamin D levels [40]. Previous studies have linked vitamin D deficiency to kidney injury, recurrent urinary tract infections, and possible alterations in nephron development, although evidence for prenatal effects remains inconclusive [52,53]. In contrast, no statistically significant association between vitamin D status and eGFR was detected in this preschool-aged cohort, in which kidney function was uniformly within the normal range and the analytic subsample was modest (*n* = 102).

Vitamin D may influence insulin sensitivity by modulating insulin receptor expression and peripheral glucose uptake [41,49]. Although some clinical studies have linked vitamin D deficiency to pancreatic β-cell dysfunction and diabetes risk [49,54], meta-analyses have reported inconsistent effects on glucose metabolism [55,56]. Consistent with the heterogeneous findings from meta-analyses, serum 25(OH)D levels were not associated with insulin resistance (measured via HOMA-IR). The lack of association may partly reflect the relatively low prevalence of insulin resistance in this healthy preschool-aged population, which may have limited the statistical power to detect modest metabolic effects. Much of the existing evidence linking vitamin D to glucose homeostasis and insulin sensitivity is drawn from populations with diabetes or established metabolic disease; the null finding in this generally healthy cohort is consistent with an effect that may be specific to, or more readily detectable in, higher-risk populations.

Vitamin D has been implicated in brain development through its regulatory effects on neural cell proliferation, differentiation, and survival during early life [57]. In the present study, in which cognitive assessment was performed in preterm children only, no statistically significant associations were detected between serum 25(OH)D concentrations and cognitive function at preschool age, consistent with a prior cohort study demonstrating similar null findings [18]. Although preterm survivors are theoretically at elevated risk of cognitive impairment, no association was detected in this subsample (*n* = 137), in which cognitive scores clustered within the normal range.

Our results demonstrated significant associations of sun exposure, physical activity, and supplementation with circulating 25(OH)D concentrations, highlighting the relevance of modifiable lifestyle factors to vitamin D status during early childhood. The association with sun exposure is biologically plausible and reinforces the central role of ultraviolet-B radiation in endogenous vitamin D synthesis. Physical activity may also reflect greater outdoor exposure and incidental sunlight [15]. These two exposures were strongly correlated in our cohort (r = 0.747), as expected given that outdoor activity is itself a source of ultraviolet exposure. The absence of a detected seasonal difference in serum 25(OH)D concentrations within this cohort (*p* = 0.632) is compatible with the observed associations with sun exposure and physical activity reflecting individual behavioral differences, although limited power to detect seasonal variation cannot be excluded. In a subtropical setting with high ambient ultraviolet exposure year-round, individual behavioral variation in outdoor time may be a stronger determinant of 25(OH)D status than calendar season; this does not represent a contradiction between the seasonal and sun exposure analyses, but reflects different levels of comparison (between-season group means versus between-individual behavioral variation). These findings describe an observed cross-sectional association between reported sun exposure and serum 25(OH)D concentration and should not be interpreted as a recommendation to increase sun exposure, given the well-established skin cancer risk associated with ultraviolet exposure.

Despite the high prevalence of deficiency, international guidelines remain inconsistent; the American Academy of Pediatrics recommends 400 IU daily [58], whereas ESPGHAN provides no specific recommendations for older children because of heterogeneous trial evidence and uncertainty regarding clinical thresholds [59]. This inconsistency extends to the definition of sufficiency itself: some authorities regard 20 ng/mL as adequate for most children, whereas the 30 ng/mL threshold used in our primary classification reflects a more conservative standard. Our findings were materially unchanged under either definition (Table S5). Although randomized controlled trials have demonstrated that supplementation reliably increases serum 25(OH)D concentrations, evidence that such increases translate into improved clinical outcomes in young children remains limited [3]. Collectively, these findings indicate that sun exposure, physical activity, and supplementation were cross-sectionally associated with higher 25(OH)D concentrations in this cohort; as an exploratory analysis restricted to the second cohort, they should be interpreted as hypothesis-generating. These correlations were modest in magnitude, indicating that physical activity, sun exposure, and supplementation account for only a limited proportion of the variance in serum 25(OH)D; other unmeasured factors likely explain most of the remaining variation. The supplementation estimate is based on only 12 supplemented children and is correspondingly imprecise, as reflected in the wide confidence interval. Although users and non-users were comparable on all measured characteristics (Table S6), supplementation was self-selected rather than assigned, and unmeasured differences in prior diagnosis of deficiency, parental health awareness, socioeconomic position, or frequency of clinical follow-up may have contributed to the observed difference.

A major strength of this study was the comprehensive multisystem assessment, which enabled evaluation of the associations between vitamin D status and a broad range of health outcomes rather than a single organ system. Nevertheless, several limitations should be acknowledged.

First, this single-center study included preterm and term children aged 5–6 years, which may limit the generalizability of the findings to other populations, age groups, or geographic regions. Taiwan’s subtropical climate provides higher ambient ultraviolet exposure than temperate or high-latitude regions for most of the year, and participants were drawn from a structured preterm follow-up program with organized clinical monitoring; findings may not generalize to populations with pronounced seasonal ultraviolet variation or to preterm survivors without comparable follow-up infrastructure. The recruitment asymmetry between the preterm arm (invited from the preterm follow-up program) and the term-control arm (recruited opportunistically from outpatient clinics and inpatient wards) is a further potential source of selection bias in the preterm–term comparison. However, enrolled and non-enrolled preterm children did not differ significantly in any perinatal or neonatal characteristic examined (Table S1), suggesting that selection within the preterm arm was unlikely to have introduced substantial bias. Term-born controls were also older at assessment than preterm children (5.9 vs. 5.5 years); age was adjusted for in the statistical models, but this difference should be considered when interpreting age-sensitive outcomes. Second, the study population primarily consisted of generally healthy survivors, potentially reducing variability in clinical outcomes and limiting detection of modest associations. Third, given the number of outcomes assessed, the findings should be interpreted as exploratory and hypothesis-generating despite the application of false discovery rate correction. Fourth, both type I and type II errors remain possible, particularly in subgroup analyses with small sample sizes, including the vitamin D supplementation subgroup (*n* = 12). All analyses were complete-case; differences in analytic sample size across outcome domains arose from the cohort-specific assessment protocols rather than from item-level missing data, and imputation was therefore not undertaken. Fifth, several potentially relevant covariates were not recorded in the original cohort protocols, including socioeconomic status, parental education, home environment, feeding history, early developmental intervention, prior diagnosis of vitamin D deficiency, parental health awareness, detailed dietary intake, indoor air quality, and secondhand smoke exposure. Residual confounding therefore cannot be excluded—particularly for the cognitive analyses, which are strongly influenced by family and environmental factors, and for the supplementation comparison, where confounding by indication and healthy-user bias remains possible. Sixth, measures of sun exposure and physical activity were based on parental reports rather than objective measures, such as ultraviolet radiation dosimetry or accelerometry, which may have introduced recall bias and resulted in either overestimation or underestimation of the true associations with serum 25(OH)D concentrations. The questionnaire also did not distinguish indoor from outdoor physical activity, nor did it record time of day, area of exposed skin, sunscreen use, clothing, weather conditions, or ambient ultraviolet index—all of which modify the relationship between reported outdoor time and cutaneous vitamin D synthesis. These items were not drawn from a previously validated instrument, and the exact item wording is not available for external validation. Seventh, although bone mineral outcomes were evaluated, parathyroid hormone concentrations, dietary calcium intake, and fracture history were not available, and the DXA reference database used to derive BMD z-scores was not derived from a Taiwanese pediatric population. Finally, restricted cubic spline or other flexible modeling of a potential non-linear dose–response relationship was not pursued; a threshold or non-linear relationship between 25(OH)D and the outcomes evaluated cannot be excluded. Despite these limitations, the findings provide a useful basis for future longitudinal studies incorporating repeated 25(OH)D measurements to evaluate temporal associations with multisystem health outcomes.

## 5. Conclusions

In conclusion, vitamin D insufficiency and deficiency were prevalent among preterm and term Taiwanese children aged 5–6 years. However, no statistically significant associations were detected between serum 25(OH)D concentrations and the multisystem health measures evaluated, including growth, body composition, bone mineral density, cardiopulmonary function, renal function, metabolic markers, or cognitive function measures; the precision of several estimates was limited by small outcome-specific subsamples. The present findings alone are insufficient to inform recommendations regarding universal vitamin D screening or supplementation in this population. In contrast, sun exposure, physical activity, and supplementation were associated with higher serum 25(OH)D concentrations; these analyses were exploratory, based on parental report, and available only in the second cohort. Future studies should incorporate repeated 25(OH)D measurements across developmental stages, prospectively defined outcomes, objective measures of ultraviolet exposure and physical activity, and more comprehensive confounder adjustment, to examine whether vitamin D status is prospectively associated with multisystem health measures in high-risk populations such as VLBW preterm survivors.

## Figures and Tables

**Table 1:** Serum 25(OH)D Levels in Preschool Children Across Selected Subgroups.

	*n*	25 (OH)D Level (ng/mL)	*p*-Value
**Gestational age** Term Children Preterm Children	69 144	22.1 ± 6.3 23.9 ± 6.4	0.053
**Sex** Boy Girl	108 105	24.1 ± 7.0 22.6 ± 5.7	0.107
**Birth weight category** Appropriate for gestational age Small for gestational age	173 40	23.3 ± 6.5 23.8 ± 6.0	0.630
**BMI level** Normal weight Underweight Overweight/obesity	155 31 27	22.8 ± 6.2 25.5 ± 7.0 24.0 ± 6.6	0.089
Vitamin D supplementation * Yes No	12 90	27.6 ± 5.8 21.8 ± 6.8	**0.006**

BMI: body mass index. * Data on vitamin D supplementation were available only from the second cohort (*n* = 102).

**Table 2:** Comparison of Health Outcomes Across Vitamin D Categories.

	Sufficiency	Insufficiency	Deficiency	Unadjusted *p*-Value	Covariate-Adjusted *p*-Value	Adjusted Mean Difference (95% CI) Sufficiency vs. Deficiency
**Basic characteristics**	**N = 38**	**N = 108**	**N = 67**			
Gestational age (weeks)	30.8 ± 4.9	31.7 ± 5.2	32.7 ± 5.4	0.174	–	–
Birth weight (g)	1536 ± 904	1635 ± 962	1901 ± 1022	0.110	–	–
Preterm (n/N, %)	29/38 (76.3%)	76/108 (70.4%)	39/67 (58.2%)	0.111	–	–
Small for gestational age (n/N, %)	7/38 (18.4%)	23/108 (21.3%)	10/67 (14.9%)	0.576	–	–
Boys (n/N, %)	26/38 (68.4%)	46/108 (42.6%)	36/67 (53.7%)	0.020	–	–
Age (year) at examination	5.5 ± 0.5	5.6 ± 0.5	5.8 ± 0.6	0.071	–	–
**Anthropometry**	**N = 38**	**N = 108**	**N = 67**			
Height z-score	−0.40 ± 1.09	−0.22 ± 0.89	−0.09 ± 0.91	0.261	0.480	−0.214 (−0.567, 0.140)
Weight z-score	−0.75 ± 1.46	−0.53 ± 1.18	−0.40 ± 1.09	0.371	0.536	−0.235 (−0.695, 0.224)
BMI z-score	−0.72 ± 1.60	−0.56 ± 1.53	−0.50 ± 1.22	0.748	0.856	−0.124 (−0.703, 0.455)
Overweight/obesity (n/N, %)	4/38 (10.5%)	18/108 (16.7%)	5/67 (7.5%)	0.187	0.183	–
	*Insufficiency vs. Deficiency: OR 2.56 (95% CI 0.88–7.47), p = 0.085* */S* *ufficiency vs. Deficiency: OR 1.66 (95% CI 0.40–6.90), p = 0.487*					
**Body composition**	**N = 14**	**N = 46**	**N = 36**			
Total lean mass index (g/m^2^)	10,254 ± 1549	10,091 ± 828	9984 ± 734	0.647	0.688	233 (−336, 803)
Total fat mass index (g/m^2^)	5002 ± 1922	4632 ± 1228	4602 ± 867	0.562	0.488	487 (−337, 1311)
Trunk fat (%)	24.4 ± 6.2	24.6 ± 5.0	24.7 ± 5.2	0.977	0.848	−0.21 (−3.32, 2.90)
Total body fat (%)	29.6 ± 5.1	29.8 ± 4.7	29.9 ± 4.6	0.980	0.786	−0.10 (−2.83, 2.63)
**Bone mineral outcomes**	**N = 14**	**N = 46**	**N = 36**			
TBLH BMC (g)	385.2 ± 67.7	400.1 ± 47.7	424.7 ± 61.7	0.047	0.178	−23.49 (−57.63, 10.66)
TBLH BMD (g/cm^2^)	0.479 ± 0.064	0.477 ± 0.032	0.489 ± 0.040	0.433	0.838	−0.003 (−0.028, 0.022)
TBLH BMD z-score	−1.03 ± 1.91	−1.28 ± 0.85	−1.02 ± 0.86	0.514	0.638	0.062 (−0.616, 0.740)
**Lung function**	**N = 24**	**N = 61**	**N = 27**			
FEV1 z-score	−0.34 ± 1.20	−0.72 ± 1.16	−0.26 ± 1.24	0.117	0.214	0.119 (−0.495, 0.734)
FVC z-score	−0.32 ± 1.21	−0.64 ± 1.25	−0.28 ± 1.09	0.247	0.398	0.126 (−0.529, 0.781)
FEV1/FVC z-score	−0.01 ± 0.94	−0.16 ± 1.13	0.09 ± 1.21	0.583	0.651	−0.022 (−0.610, 0.566)
FEF25−75 z-score	−0.56 ± 1.06	−0.83 ± 1.23	−0.48 ± 1.38	0.352	0.504	0.099 (−0.531, 0.730)
**Cardiovascular outcomes**	**N = 38**	**N = 108**	**N = 67**			
Systolic BP (mmHg)	103.7 ± 8.7	104.0 ± 11.6	106.3 ± 8.8	0.295	0.630	−1.62 (−5.76, 2.52)
Diastolic BP (mmHg)	63.5 ± 8.0	64.8 ± 8.9	64.8 ± 8.1	0.680	0.205	−2.63 (−5.72, 0.46)
**Echocardiographic results**	**N = 28**	**N = 59**	**N = 27**			
AoV annulus (mm)	11.8 ± 1.6	12.1 ± 1.6	12.1 ± 1.2	0.779	0.713	−0.132 (−0.943, 0.678)
Left atrium (mm)	20.4 ± 3.0	21.7 ± 3.2	21.1 ± 3.0	0.633	0.489	−0.59 (−2.26, 1.08)
IVSd (mm)	5.3 ± 0.7	5.6 ± 0.7	5.5 ± 0.6	0.126	0.063	−0.206 (−0.573, 0.162)
LVIDd (mm)	31.7 ± 3.0	31.3 ± 2.9	32.8 ± 2.8	0.108	0.278	−0.96 (−2.51, 0.59)
LVIDs (mm)	20.1 ± 2.2	20.1 ± 1.9	20.9 ± 2.1	0.197	0.428	−0.63 (−1.69, 0.42)
LVEDV (ml)	40.5 ± 8.8	39.3 ± 8.6	43.7 ± 9.0	0.107	0.280	−2.84 (−7.45, 1.78)
LVESV (ml)	13.0 ± 3.6	13.1 ± 2.9	14.6 ± 3.4	0.083	0.217	−1.29 (−2.91, 0.33)
LVM (g)	36.9 ± 9.1	39.2 ± 8.9	40.1 ± 8.0	0.204	0.127	−3.83 (−8.49, 0.84)
Stroke volume (ml)	27.6 ± 6.5	26.2 ± 6.9	29.1 ± 6.8	0.221	0.434	−1.55 (−5.17, 2.07)
Ejection fraction (%)	67.7 ± 5.7	66.4 ± 5.9	66.4 ± 5.3	0.614	0.737	0.82 (−2.16, 3.81)
Shortening fraction (%)	36.7 ± 4.5	35.5 ± 4.6	36.1 ± 4.5	0.746	0.792	0.03 (−2.42, 2.47)
LV global longitudinal strain (%)	−22.1 ± 1.5	−21.6 ± 1.7	−22.0 ± 2.1	0.247	0.222	−0.46 (−1.34, 0.42)
LA longitudinal strain (%)	47.3 ± 8.1	46.1 ± 7.5	47.1 ± 7.7	0.367	0.241	2.62 (−1.03, 6.26)
**Kidney function**	**N = 14**	**N = 48**	**N = 40**			
BUN (mg/dL)	13.4 ± 2.1	13.2 ± 3.2	12.3 ± 2.6	0.315	0.274	0.83 (−0.92, 2.59)
Cr (mg/dL)	0.41 ± 0.07	0.41 ± 0.07	0.42 ± 0.07	0.735	0.830	−0.003 (−0.046, 0.041)
Cystatin C (mg/L)	0.95 ± 0.12	0.92 ± 0.16	0.94 ± 0.17	0.665	0.559	−0.024 (−0.115, 0.067)
eGFR-Cr (mL/min/1.73 m^2^)	98.2 ± 15.3	99.3 ± 16.8	99.3 ± 15.9	0.974	0.879	−0.43 (−10.33, 9.47)
eGFR-CysC (mL/min/1.73 m^2^)	83.8 ± 10.4	88.0 ± 15.5	86.0 ± 17.0	0.646	0.712	0.98 (−7.81, 9.77)
Plasma renin activity (ng/mL/hr)	3.47 ± 2.46	3.52 ± 2.37	3.23 ± 2.43	0.850	0.711	0.44 (−1.14, 2.01)
Kidney length, Right side (mm)	76.3 ± 8.3	75.8 ± 4.7	77.3 ± 5.2	0.433	0.614	0.18 (−3.00, 3.36)
Kidney length, Left side (mm)	78.7 ± 7.9	77.8 ± 5.4	78.7 ± 6.2	0.726	0.526	1.54 (−1.98, 5.06)
**Biochemical**	**N = 14**	**N = 48**	**N = 40**			
Albumin (g/dL)	4.7 ± 0.2	4.8 ± 0.2	4.8 ± 0.3	0.325	0.976	−0.007 (−0.147, 0.134)
Cholesterol (mg/dL)	167.4 ± 18.9	175.7 ± 25.8	172.2 ± 24.5	0.514	0.690	−1.76 (−17.33, 13.80)
Triglyceride (mg/dL)	36.5 ± 10.7	47.7 ± 22.1	50.2 ± 22.5	0.117	0.106	−0.272 (−0.526, −0.017)
LDL (mg/dL)	96.9 ± 22.1	99.8 ± 22.9	98.2 ± 22.0	0.888	0.859	−0.99 (−15.04, 13.06)
HDL (mg/dL)	60.2 ± 13.3	62.2 ± 12.8	60.8 ± 9.9	0.804	0.816	0.61 (−7.14, 8.36)
Calcium (mg/dL)	9.8 ± 0.3	9.8 ± 0.3	9.8 ± 0.3	0.883	0.717	0.059 (−0.107, 0.224)
Phosphate (mg/dL)	5.0 ± 0.5	5.1 ± 0.4	5.3 ± 0.5	0.157	0.070	−0.155 (−0.428, 0.117)
Alkaline Phosphatase (IU/L)	190.9 ± 40.3	202.2 ± 44.9	217.0 ± 56.0	0.169	0.109	−30.27 (−60.15, −0.40)
Glucose (mg/dL)	87.3 ± 5.3	87.6 ± 5.3	89.3 ± 5.9	0.320	0.333	−2.44 (−5.79, 0.91)
Insulin (uU/mL)	4.4 ± 3.5	5.3 ± 3.4	4.7 ± 2.8	0.565	0.620	−0.145 (−0.558, 0.268)
HOMA-IR index	0.96 ± 0.77	1.16 ± 0.79	1.05 ± 0.66	0.619	0.601	−0.174 (−0.609, 0.261)
Cognitive outcomes *	**N = 27**	**N = 72**	**N = 38**			
Full-Scale Intelligence Quotient (FSIQ)	90.3 ± 11.0	94.2 ± 10.5	95.7 ± 12.5	0.222	0.360	−3.96 (−9.53, 1.60)
Verbal Comprehension Index	95.0 ± 11.4	97.8 ± 11.6	99.9 ± 13.3	0.379	0.517	−3.15 (−9.07, 2.77)
Visual Spatial Index	92.0 ± 13.3	93.8 ± 12.2	93.3 ± 14.3	0.897	0.967	−0.65 (−7.29, 5.99)
Fluid Reasoning Index	92.0 ± 8.0	92.4 ± 10.4	94.2 ± 11.1	0.602	0.613	−1.09 (−6.04, 3.86)
Processing Speed Index	92.5 ± 14.1	97.7 ± 13.4	97.3 ± 14.8	0.689	0.478	−4.13 (−11.06, 2.80)
Working Memory Index	91.7 ± 15.4	94.3 ± 11.3	93.3 ± 14.1	0.309	0.946	−0.73 (−7.31, 5.85)
**Questionnaire-derived outcomes**						
Physician diagnosed asthma (n/N, %)	7/25 (28.0%)	19/62 (30.6%)	4/27 (14.8%)	0.290	0.244	–
	*Insufficiency vs. Deficiency: OR 2.75 (95% CI 0.79–9.57), p = 0.112* */* *Sufficiency vs. Deficiency: OR 1.97 (95% CI 0.47–8.28), p = 0.356*					
Lower respiratory tract infection hospitalization (n/N, %)	17/38 (44.7%)	44/108 (40.7%)	22/67 (32.8%)	0.420	0.184	–
	*Insufficiency vs. Deficiency: OR 1.66 (95% CI 0.84–3.25), p = 0.142* */* *Sufficiency vs. Deficiency: OR 2.08 (95% CI 0.88–4.94), p = 0.097*					
Physical activity (hours/week)	4.1 ± 2.3	4.2 ± 1.9	3.3 ± 1.8	0.121	**0.043**	1.20 (−0.01, 2.42)
Sun exposure (hours/week)	4.4 ± 2.3	3.5 ± 2.0	3.0 ± 3.0	0.100	**0.025**	1.81 (0.50, 3.12)
Vitamin D supplementation (n/N, %)	3/14 (21.4%)	8/48 (16.7%)	1/40 (2.5%)	0.058	**0.029**	–
	*Insufficiency vs. Deficiency: OR 10.06 (95% CI 1.06–95.48), p = 0.044* */* *Sufficiency vs. Deficiency: OR 10.48 (95% CI 0.89–123.12), p = 0.062*					

BMI, body mass index; TBLH, total body less head; BMC, bone mineral content; BMD, bone mineral density; FEV_1_, forced expiratory volume in the first second; FVC, forced vital capacity; FEF_25–75_, forced expiratory flow 25–75%; AoV, aortic valve; IVSd, interventricular septal end-diastolic dimension; LVIDd, left ventricular end-diastolic internal dimension; LVIDs, left ventricular end-systolic internal dimension; LVM, left ventricular mass; LVEDV, left ventricular end-diastolic volume; LVESV, left ventricular end-systolic volume; LV, left ventricle; LA, left atrium; BUN, blood urea nitrogen; Cr, creatinine; eGFR, estimated glomerular filtration rate; LDL, low-density lipoprotein; HDL, high-density lipoprotein; HOMA-IR, Homeostasis Model Assessment of Insulin Resistance. * Preterm children only. Adjusted *p*-value: standardized (z-score/eGFR/BMD-z) outcomes adjusted for gestational age (GA) + small for gestational age (SGA) + season; all other continuous outcomes adjusted for age + sex + GA + SGA + season; anthropometry and blood pressure additionally adjusted for cohort. Categorical outcomes (overweight/obesity, asthma, lower respiratory tract infection hospitalization, supplementation) analyzed by logistic regression with the same covariates; the adjusted *p*-value is the omnibus likelihood-ratio test for the vitamin D category term, with odds ratios (95% CI) per category shown beneath each row (reference = deficiency). Adjusted mean difference compares the sufficiency group with the deficiency group (reference), from the same covariate-adjusted model. False discovery rate q-values for all comparisons are reported in Supplementary Table S4.

## Data Availability

The data presented in this study are available from the corresponding author upon request. These data are not publicly available due to privacy and ethical restrictions involving pediatric participants.

## Supplementary Materials

The following supporting information can be downloaded at: https://www.mdpi.com/article/10.3390/nu18182994/s1, Table S1: Perinatal and neonatal characteristics of very low birth weight preterm children enrolled and not enrolled in the present vitamin D analysis; Table S2: Comparison of participant characteristics between the two source cohorts; Table S3: Effect sizes for statistically significant continuous differences between cohorts; Table S4: Benjamini–Hochberg false discovery rate correction within each outcome domain; Table S5: Sensitivity analysis using a single 20 ng/mL threshold; Table S6: Characteristics of vitamin D supplement users and non-users (Cohort 2, n = 102).

## Abbreviations

The following abbreviations are used in this manuscript:

25(OH)D	25-hydroxyvitamin D
VLBW	very low birth weight
GA	gestational age
DXA	dual-energy X-ray absorptiometry
BW	birth weight
BP	blood pressure
BMI	body mass index
FEV_1_	forced expiratory volume in one second
FVC	forced vital capacity
FEF_25–75_	forced expiratory flow between 25% and 75% of expired forced vital capacity
2DSTE	two-dimensional speckle-tracking echocardiography
BMC	bone mineral content
BMD	bone mineral density
TBLH	total body less head
LRTI	lower respiratory tract infection
eGFR	estimated glomerular filtration rate
HOMA-IR	homeostatic model assessment of insulin resistance
FSIQ	full-scale intelligence quotient
SGA	small for gestational age
ANOVA	analysis of variance
