# Supplementary material for: Vitamin D Status and Multisystem Health Outcomes at 5–6 Years in Very-Low-Birth-Weight Preterm and Term-Born Children: A Cross-Sectional Secondary Analysis of Two Prospective Cohorts
Source: Nutrients. 2026 Sep 13;18(18):2994. doi: 10.3390/nu18182994 (PMC13610414; doi:10.3390/nu18182994)
Supplement: Supplementary file 1 [file nutrients-18-02994-s001.zip › nutrients-4485323-supplementary.pdf]

Supplementary Tables S1–S6

Table S1. Perinatal and neonatal characteristics of very low birth weight preterm children enrolled and not enrolled in the present vitamin D analysis

Comparison across the full VLBW preterm sample followed in the institutional preterm follow-up program during the study period (n = 286).

Characteristic	Enrolled (n=144)	Not enrolled (n=142)	p value
Gestational age (weeks)	28.6 ± 2.7	28.5 ± 2.9	0.691
Birth weight (g)	1056 ± 256	1062 ± 263	0.843
Male sex	71/142 (50.0%)	62/141 (44.0%)	0.341
Multiple birth	26/142 (18.3%)	39/141 (27.7%)	0.067
Small for gestational age	46/142 (32.4%)	46/141 (32.6%)	1.000
Cesarean delivery	96/141 (68.1%)	108/141 (76.6%)	0.143
PROM > 18 h	48/141 (34.0%)	46/141 (32.6%)	0.900
Apgar score < 7 at 5 min	11/142 (7.7%)	10/141 (7.1%)	1.000
Maternal preeclampsia	36/141 (25.5%)	32/141 (22.7%)	0.676
Antenatal corticosteroids	117/141 (83.0%)	127/141 (90.1%)	0.116
RDS requiring surfactant	46/140 (32.9%)	46/141 (32.6%)	1.000
Chronic lung disease	73/142 (51.4%)	81/141 (57.4%)	0.340
PDA requiring treatment	47/144 (32.6%)	52/142 (36.6%)	0.535
Any sepsis	25/142 (17.6%)	22/141 (15.6%)	0.750
Severe IVH	10/142 (7.0%)	9/141 (6.4%)	1.000
Periventricular leukomalacia	6/142 (4.2%)	7/141 (5.0%)	0.785
Necrotizing enterocolitis	1/142 (0.7%)	2/141 (1.4%)	0.622
ROP requiring treatment	15/142 (10.6%)	19/141 (13.5%)	0.471

Data are mean ± SD or n/N (%).

Continuous variables: Welch t-test; categorical variables: Fisher exact test. Denominators vary slightly because of missing values for individual perinatal variables.

PROM, prolonged rupture of membranes; RDS, respiratory distress syndrome; PDA, patent ductus arteriosus; IVH, intraventricular hemorrhage; ROP, retinopathy of prematurity.

Table S2. Comparison of participant characteristics between the two source cohorts

Characteristic	Cohort 1 (n=114)	Cohort 2 (n=102)	p value
Age at assessment (years)	5.7 ± 0.6	5.6 ± 0.5	0.825
Serum 25(OH)D (ng/mL)	24.3 ± 5.9	22.5 ± 6.9	**0.038**
Gestational age (weeks)	31.1 ± 4.9	32.6 ± 5.4	**0.030**
Birth weight (g)	1522 ± 888	1877 ± 1038	**0.008**
Preterm	86/114 (75.4%)	61/102 (59.8%)	**0.019**
Small for gestational age	24/114 (21.1%)	17/102 (16.7%)	0.488
Boys	58/114 (50.9%)	52/102 (51.0%)	1.000
**Season of blood sampling**			**0.015**
Spring	27 (23.7%)	28 (27.5%)	
Summer	42 (36.8%)	19 (18.6%)	
Autumn	25 (21.9%)	37 (36.3%)	
Winter	20 (17.5%)	18 (17.6%)	
**Year of assessment**			**<0.001**
2017	22 (19.3%)	0 (0.0%)	
2018	59 (51.8%)	0 (0.0%)	
2019	30 (26.3%)	0 (0.0%)	
2020	1 (0.9%)	5 (4.9%)	
2021	2 (1.8%)	33 (32.4%)	
2022	0 (0.0%)	64 (62.7%)	

Data are mean ± SD or n/N (%).

Continuous variables: Welch t-test; categorical variables: Fisher exact test (2×2) or chi-square test. Three preterm participants enrolled in both cohorts are represented in both columns; a sensitivity analysis excluding them (Cohort 1 n=111, Cohort 2 n=99) yielded materially unchanged results. Vitamin D supplementation was recorded only in Cohort 2 and could not be compared. Year of assessment is shown descriptively; as with cohort membership, it was not entered as a covariate because each outcome domain is analyzed within, not across, cohorts. Bold/shaded = p<0.05.

Table S3. Effect sizes for statistically significant continuous differences between cohorts

Purpose: to distinguish statistical significance (driven by sample size) from clinical significance (magnitude of difference).

Interpretation: Cohen's d < 0.2 negligible; 0.2–0.5 small; 0.5–0.8 medium; ≥0.8 large.

Variable	Unit	Cohort 1 (n=114)	Cohort 2 (n=102)	Mean difference	% difference	Cohen's d	Magnitude
Serum 25(OH)D	ng/mL	24.3 ± 5.9	22.5 ± 6.9	1.84	7.6%	0.287	Small
Gestational age	weeks	31.1 ± 4.9	32.6 ± 5.4	-1.55	5.0%	-0.299	Small
Birth weight	g	1521 ± 888	1877 ± 1038	-355.83	23.4%	-0.370	Small
Age at assessment	years	5.7 ± 0.6	5.6 ± 0.5	0.02	0.3%	0.030	Negligible

All four differences fall in the negligible-to-small range despite three reaching statistical significance. The 1.84 ng/mL difference in mean 25(OH)D does not cross the deficiency (<20) / insufficiency (20–30) / sufficiency (>30 ng/mL) category boundaries; both cohort means fall within the insufficiency band.

Table S4. Benjamini–Hochberg false discovery rate correction within each outcome domain

FDR correction was applied separately within each outcome domain, and separately for Table 2 (categorical 25(OH)D, omnibus test) and Table 3 (continuous 25(OH)D, covariate-adjusted regression/partial correlation).

Domain / Table	Variable	Adjusted p-value	FDR q-value
**Table 2 — Anthropometry/growth (n=4 tests)**			
	Height z-score	0.480	0.715
	Weight z-score	0.536	0.715
	Overweight/obesity	0.183	0.715
	BMI z-score	0.856	0.856
**Table 2 — Biochemical/renal (n=19 tests)**			
	Triglyceride	0.106	0.690
	Phosphate	0.070	0.690
	Alkaline phosphatase	0.109	0.690
	Cholesterol	0.690	0.927
	LDL	0.859	0.927
	HDL	0.816	0.927
	Calcium	0.717	0.927
	Glucose	0.333	0.927
	Insulin	0.620	0.927
	HOMA-IR	0.601	0.927
	BUN	0.274	0.927
	Creatinine	0.830	0.927
	Cystatin C	0.559	0.927
	eGFR-Cr	0.879	0.927
	eGFR-CysC	0.712	0.927
	Plasma renin activity	0.711	0.927
	Kidney length R	0.614	0.927
	Kidney length L	0.526	0.927
	Albumin	0.976	0.976
**Table 2 — Body composition (n=4 tests)**			
	Total lean mass index	0.688	0.848

Domain / Table	Variable	Adjusted p-value	FDR q-value
	Total fat mass index	0.488	0.848
	Trunk fat %	0.848	0.848
	Total body fat %	0.786	0.848
**Table 2 — Bone (n=3 tests)**			
	TBLH BMC	0.178	0.533
	TBLH BMD	0.838	0.838
	TBLH BMD Z-score	0.638	0.838
**Table 2 — Cardiopulmonary (n=19 tests)**			
	FEV1 z-score	0.214	0.592
	Diastolic BP	0.205	0.592
	IVSd	0.063	0.592
	LVIDd	0.278	0.592
	LVEDV	0.280	0.592
	LVESV	0.217	0.592
	LV mass	0.127	0.592
	LV global longitudinal strain	0.222	0.592
	LA longitudinal strain	0.241	0.592
	FVC z-score	0.398	0.685
	FEF25-75 z-score	0.504	0.685
	Left atrium	0.489	0.685
	LVIDs	0.428	0.685
	Stroke volume	0.434	0.685
	FEV1/FVC z-score	0.651	0.773
	Systolic BP	0.630	0.773
	AoV annulus	0.713	0.778
	Ejection fraction	0.737	0.778
	Shortening fraction	0.792	0.792
**Table 2 — Cognitive (n=6 tests)**			
	FSIQ	0.360	0.919
	VCI	0.517	0.919

Domain / Table	Variable	Adjusted p-value	FDR q-value
	FRI	0.613	0.919
	PSI	0.478	0.919
	VSI	0.967	0.967
	WMI	0.946	0.967
**Table 2 — Questionnaire (n=5 tests)**			
	Physical activity	0.043	0.071
	Sun exposure	0.025	0.071
	Vitamin D supplementation	0.029	0.071
	LRTI hospitalization	0.184	0.230
	Physician-diagnosed asthma	0.244	0.244
**Table 3 — Anthropometry/growth (n=3 tests)**			
	Height z-score	0.282	0.424
	Weight z-score	0.170	0.424
	BMI z-score	0.492	0.492
**Table 3 — Biochemical/renal (n=9 tests)**			
	Calcium	0.285	0.566
	Phosphate	0.319	0.566
	Alkaline phosphatase	0.104	0.566
	HOMA-IR	0.360	0.566
	Cystatin C	0.377	0.566
	eGFR-CysC	0.274	0.566
	Insulin	0.486	0.624
	eGFR-Cr	0.655	0.736
	Creatinine	0.959	0.959
**Table 3 — Body composition (n=4 tests)**			
	Total lean mass index	0.643	0.643
	Total fat mass index	0.562	0.643
	Trunk fat %	0.370	0.643
	Total body fat %	0.300	0.643
**Table 3 — Bone (n=3 tests)**			

Domain / Table	Variable	Adjusted p-value	FDR q-value
	TBLH BMC	0.118	0.354
	TBLH BMD	0.987	0.987
	TBLH BMD Z-score	0.820	0.987
**Table 3 — Cardiopulmonary (n=12 tests)**			
	FEV1/FVC z-score	0.160	0.384
	Diastolic BP	0.159	0.384
	LVEDV	0.072	0.384
	LVESV	0.122	0.384
	Stroke volume	0.094	0.384
	FVC z-score	0.284	0.487
	Systolic BP	0.275	0.487
	FEV1 z-score	0.377	0.540
	Shortening fraction	0.405	0.540
	LA longitudinal strain	0.465	0.558
	FEF25-75 z-score	0.915	0.923
	LV global longitudinal strain	0.923	0.923
**Table 3 — Cognitive (n=1 tests)**			
	FSIQ	0.241	0.241
**Table 3 — Questionnaire (n=3 tests)**			
	Sun exposure	<0.001	**0.002**
	Physical activity	0.002	**0.003**
	Vitamin D supplementation	0.009	**0.009**

Adjusted p-value = covariate-adjusted p-value as reported in Table 2 or Table 3.

Plasma renin activity was analyzed using the Kruskal–Wallis test rather than covariate-adjusted ANCOVA.

Bold/shaded = q<0.05.

Table S5. Sensitivity analysis using a single 20 ng/mL threshold

Adjusted mean difference comparing children with 25(OH)D ≥20 ng/mL (n=146) with those <20 ng/mL (n=67, reference).

Outcome	N	25(OH)D ≥20 ng/mL (n)	25(OH)D <20 ng/mL (n)	Adjusted difference	95% CI	*P* value
Height z-score	213	146	67	-0.093	(-0.350, 0.164)	0.477
Weight z-score	213	146	67	-0.068	(-0.403, 0.266)	0.687
BMI z-score	213	146	67	-0.012	(-0.433, 0.408)	0.954
Total lean mass index	96	60	36	144	(-231, 519)	0.446
Total fat mass index	96	60	36	161	(-384, 706)	0.559
TBLH BMC (g)	96	60	36	-21.24	(-43.70, 1.21)	0.063
TBLH BMD z-score	96	60	36	-0.130	(-0.582, 0.321)	0.567
FEV1 z-score	112	85	27	-0.185	(-0.673, 0.303)	0.454
FVC z-score	112	85	27	-0.131	(-0.649, 0.386)	0.616
FEV1/FVC z-score	112	85	27	-0.147	(-0.609, 0.316)	0.530
FEF25-75 z-score	112	85	27	-0.113	(-0.610, 0.385)	0.654
Systolic BP (mmHg)	213	146	67	-1.45	(-4.45, 1.54)	0.340
Diastolic BP (mmHg)	213	146	67	-0.89	(-3.15, 1.36)	0.435
LVEDV (ml)	114	87	27	-2.96	(-6.61, 0.69)	0.110
LV mass (g)	114	87	27	-0.99	(-4.75, 2.76)	0.601
LV GLS (%)	114	87	27	0.014	(-0.692, 0.721)	0.968
Triglyceride [log]	102	62	40	-0.133	(-0.298, 0.031)	0.111
HOMA-IR [log]	102	62	40	-0.004	(-0.283, 0.275)	0.975
Calcium (mg/dL)	102	62	40	0.007	(-0.099, 0.113)	0.897
Phosphate (mg/dL)	102	62	40	-0.202	(-0.377, -0.028)	**0.023**
ALP (IU/L)	102	62	40	-17.67	(-36.87, 1.53)	0.071
eGFR-Cr	102	62	40	1.06	(-5.33, 7.45)	0.744
Cystatin C (mg/L)	102	62	40	-0.031	(-0.089, 0.027)	0.288
FSIQ	137	99	38	-2.66	(-6.87, 1.55)	0.214
Physical activity (h/wk)	102	62	40	0.98	(0.21, 1.76)	**0.013**
Sun exposure (h/wk)	102	62	40	0.87	(0.01, 1.72)	**0.046**

Covariate adjustment as in the primary analysis: standardized (z-score/eGFR/BMD-z) outcomes adjusted for gestational age + SGA + season; all other outcomes adjusted for age + sex + gestational age + SGA + season; blood pressure additionally adjusted

for cohort.

Bold/shaded = p<0.05.

Table S6. Characteristics of vitamin D supplement users and non-users (Cohort 2, n = 102)

Characteristic	Users (n=12)	Non-users (n=90)	p value
Age at assessment (years)	5.6 ± 0.7	5.6 ± 0.5	0.952
Gestational age (weeks)	33.0 ± 4.7	32.6 ± 5.6	0.775
Birth weight (g)	1928 ± 1160	1871 ± 1028	0.874
BMI z-score	-1.14 ± 1.84	-0.55 ± 1.38	0.305
Serum 25(OH)D (ng/mL)	27.6 ± 5.8	21.8 ± 6.8	**0.006**
Physical activity (h/wk)	4.5 ± 1.5	3.7 ± 2.0	0.134
Sun exposure (h/wk)	4.5 ± 1.7	3.3 ± 2.1	**0.048**
Preterm	7/12 (58.3%)	54/90 (60.0%)	1.0
Boys	7/12 (58.3%)	45/90 (50.0%)	0.761
Small for gestational age	3/12 (25.0%)	14/90 (15.6%)	0.417
Season distribution (chi-square)			0.126

Data are mean ± SD or n/N (%).

Continuous variables: Welch t-test; categorical variables: Fisher exact test; season distribution: chi-square test.

Supplementation was defined as intake of ≥400 IU/day for at least 6 months and was recorded as a binary variable.

Bold/shaded = p<0.05.
